# 2-{[2-(1-Methyl-2,2-dioxo-3,4-dihydro-1*H*-2λ^6^,1-benzothia­zin-4-yl­idene)hydrazin-1-yl­idene]meth­yl}phenol

**DOI:** 10.1107/S1600536812034101

**Published:** 2012-08-04

**Authors:** Muhammad Shafiq, William T. A. Harrison, Islam Ullah Khan, Iftikhar Hussain Bukhari, Tanveer Hussain Bokhari

**Affiliations:** a Department of Chemistry, Government College University, Faisalabad 38000, Pakistan; b Department of Chemistry, University of Aberdeen, Meston Walk, Aberdeen AB24 3UE, Scotland; c Materials Chemistry Laboratory, Department of Chemistry, Government College University, Lahore, Pakistan

## Abstract

In the title compound, C_16_H_15_N_3_O_3_S, the dihedral angle between the aromatic rings is 8.18 (11)° and the C=N—N=C torsion angle is 178.59 (14)°. The conformation of the thia­zine ring is an envelope, with the S atom displaced by 0.8157 (18) Å from the mean plane of the other five atoms (r.m.s. deviation = 0.045 Å). An intra­molecular O—H⋯N hydrogen bond closes an *S*(6) ring. In the crystal, weak C—H⋯O inter­actions link the mol­ecules, with all three O atoms acting as acceptors.

## Related literature

For the synthesis and biological activity of the title compound and related materials, see: Shafiq *et al.* (2011 ▶).
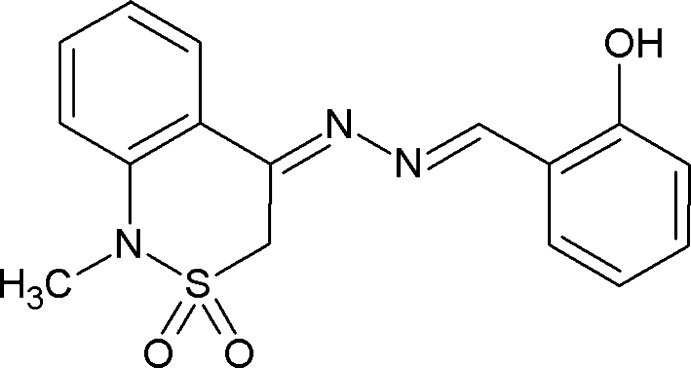


## Experimental

### 

#### Crystal data

C_16_H_15_N_3_O_3_S
*M* *_r_* = 329.37
Monoclinic, 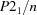

*a* = 6.5530 (2) Å
*b* = 15.8719 (5) Å
*c* = 14.5804 (4) Å
β = 91.147 (1)°
*V* = 1516.18 (8) Å^3^
*Z* = 4
Mo *K*α radiation
μ = 0.23 mm^−1^
*T* = 296 K
0.40 × 0.05 × 0.05 mm

#### Data collection

Bruker APEXII CCD diffractometer
14391 measured reflections
3778 independent reflections
3019 reflections with *I* > 2σ(*I*)
*R*_int_ = 0.020

#### Refinement

*R*[*F* ^2^ > 2σ(*F* ^2^)] = 0.039
*wR*(*F* ^2^) = 0.114
*S* = 1.03
3778 reflections
212 parameters
H atoms treated by a mixture of independent and constrained refinement
Δρ_max_ = 0.32 e Å^−3^
Δρ_min_ = −0.28 e Å^−3^

### 

Data collection: *APEX2* (Bruker, 2007 ▶); cell refinement: *SAINT* (Bruker, 2007 ▶); data reduction: *SAINT*; program(s) used to solve structure: *SHELXS97* (Sheldrick, 2008 ▶); program(s) used to refine structure: *SHELXL97* (Sheldrick, 2008 ▶); molecular graphics: *PLATON* (Spek, 2009 ▶); software used to prepare material for publication: *ORTEP-3* (Farrugia, 1997 ▶).

## Supplementary Material

Crystal structure: contains datablock(s) global, I. DOI: 10.1107/S1600536812034101/bt5989sup1.cif

Structure factors: contains datablock(s) I. DOI: 10.1107/S1600536812034101/bt5989Isup2.hkl

Supplementary material file. DOI: 10.1107/S1600536812034101/bt5989Isup3.cml

Additional supplementary materials:  crystallographic information; 3D view; checkCIF report

## Figures and Tables

**Table 1:** Hydrogen-bond geometry (Å, °)

*D*—H⋯*A*	*D*—H	H⋯*A*	*D*⋯*A*	*D*—H⋯*A*
O3—H3⋯N3	0.85 (2)	1.89 (2)	2.6574 (18)	151 (2)
C1—H1*B*⋯O3^i^	0.96	2.59	3.534 (3)	166
C3—H3*A*⋯O1^ii^	0.93	2.60	3.396 (2)	144
C9—H9*A*⋯O2^i^	0.97	2.44	3.138 (2)	129
C16—H16⋯O1^iii^	0.93	2.60	3.440 (2)	151

Symmetry codes: (i) 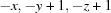; (ii) 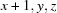; (iii) 
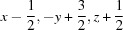
.

**Fig. 1.**
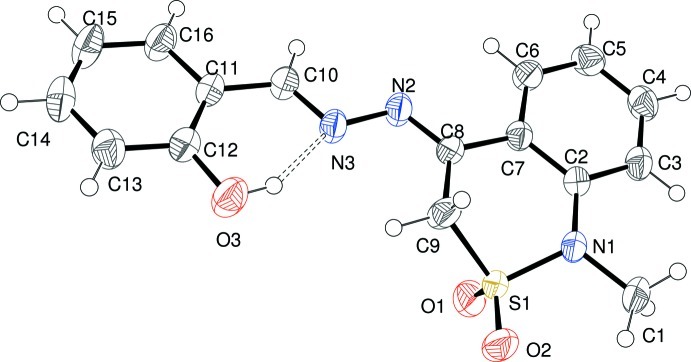
The molecular structure of (I), showing displacement ellipsoids at the 50% probability level. The hydrogen bond is shown as a double-dashed line.

## supplementary crystallographic information

### Experimental

In the synthesis of title compound, 4-hydrazinylidene-1- methyl-3H-2λ^6^,1-benzothiazine-2,2-dione was subjected to react with 2-hydroxy benzaldehyde according to literature procedure ((Shafiq *et al.* (2011)). The product obtained was then recrystallized in ethylacetate under slow evaporation to obtain single crystals suitable for X-ray diffraction.

### Refinement

The O-bond H atom was located in a difference map and its position was freely refined. The C-bound H-atoms were placed in calculated positions (C—H = 0.93–0.97 Å) and refined as riding. The methyl group was allowed to rotate, but not to tip, to best fit the electron density. The constraint *U*_iso_(H) = 1.2*U*_eq_(C,O) or 1.5*U*_eq_(methyl C) was applied.

### Crystal data

**Table:**

C_16_H_15_N_3_O_3_S	*F*(000) = 688
*M**_r_* = 329.37	*D*_x_ = 1.443 Mg m^−^^3^
Monoclinic, *P*2_1_/*n*	Mo *K*α radiation, λ = 0.71073 Å
Hall symbol: -P 2yn	Cell parameters from 6831 reflections
*a* = 6.5530 (2) Å	θ = 2.8–28.2°
*b* = 15.8719 (5) Å	µ = 0.23 mm^−^^1^
*c* = 14.5804 (4) Å	*T* = 296 K
β = 91.147 (1)°	Needle, yellow
*V* = 1516.18 (8) Å^3^	0.40 × 0.05 × 0.05 mm
*Z* = 4	

### Data collection

**Table:**

Bruker APEXII CCD diffractometer	3019 reflections with *I* > 2σ(*I*)
Radiation source: fine-focus sealed tube	*R*_int_ = 0.020
Graphite monochromator	θ_max_ = 28.4°, θ_min_ = 2.6°
φ and ω scans	*h* = −8→8
14391 measured reflections	*k* = −21→20
3778 independent reflections	*l* = −19→19

### Refinement

**Table:**

Refinement on *F*^2^	Primary atom site location: structure-invariant direct methods
Least-squares matrix: full	Secondary atom site location: difference Fourier map
*R*[*F*^2^ > 2σ(*F*^2^)] = 0.039	Hydrogen site location: inferred from neighbouring sites
*wR*(*F*^2^) = 0.114	H atoms treated by a mixture of independent and constrained refinement
*S* = 1.03	*w* = 1/[σ^2^(*F*_o_^2^) + (0.0577*P*)^2^ + 0.5143*P*] where *P* = (*F*_o_^2^ + 2*F*_c_^2^)/3
3778 reflections	(Δ/σ)_max_ = 0.001
212 parameters	Δρ_max_ = 0.32 e Å^−^^3^
0 restraints	Δρ_min_ = −0.28 e Å^−^^3^

### Special details

**Table:**

Geometry. All e.s.d.'s (except the e.s.d. in the dihedral angle between two l.s. planes) are estimated using the full covariance matrix. The cell e.s.d.'s are taken into account individually in the estimation of e.s.d.'s in distances, angles and torsion angles; correlations between e.s.d.'s in cell parameters are only used when they are defined by crystal symmetry. An approximate (isotropic) treatment of cell e.s.d.'s is used for estimating e.s.d.'s involving l.s. planes.
Refinement. Refinement of *F*^2^ against ALL reflections. The weighted *R*-factor *wR* and goodness of fit *S* are based on *F*^2^, conventional *R*-factors *R* are based on *F*, with *F* set to zero for negative *F*^2^. The threshold expression of *F*^2^ > σ(*F*^2^) is used only for calculating *R*-factors(gt) *etc*. and is not relevant to the choice of reflections for refinement. *R*-factors based on *F*^2^ are statistically about twice as large as those based on *F*, and *R*- factors based on ALL data will be even larger.

### Fractional atomic coordinates and isotropic or equivalent isotropic displacement parameters (Å^2^)

**Table:**

	*x*	*y*	*z*	*U*_iso_*/*U*_eq_	
C1	0.5578 (3)	0.63906 (15)	0.45564 (14)	0.0583 (5)	
H1A	0.5592	0.6923	0.4245	0.087*	
H1B	0.5077	0.5963	0.4144	0.087*	
H1C	0.6939	0.6250	0.4760	0.087*	
C2	0.4661 (2)	0.70248 (9)	0.60704 (10)	0.0323 (3)	
C3	0.6391 (2)	0.75336 (11)	0.60255 (12)	0.0418 (4)	
H3A	0.7228	0.7498	0.5519	0.050*	
C4	0.6875 (3)	0.80886 (11)	0.67218 (12)	0.0453 (4)	
H4	0.8039	0.8420	0.6683	0.054*	
C5	0.5651 (3)	0.81578 (11)	0.74765 (12)	0.0436 (4)	
H5	0.5973	0.8538	0.7942	0.052*	
C6	0.3944 (2)	0.76554 (10)	0.75302 (11)	0.0384 (3)	
H6	0.3127	0.7699	0.8042	0.046*	
C7	0.3401 (2)	0.70828 (9)	0.68407 (10)	0.0318 (3)	
C8	0.1578 (2)	0.65501 (9)	0.69491 (10)	0.0323 (3)	
C9	0.1241 (3)	0.58446 (12)	0.62738 (11)	0.0470 (4)	
H9A	−0.0177	0.5670	0.6280	0.056*	
H9B	0.2080	0.5366	0.6450	0.056*	
C10	−0.2398 (2)	0.63187 (10)	0.83713 (11)	0.0375 (3)	
H10	−0.2025	0.6761	0.8758	0.045*	
C11	−0.4218 (2)	0.58389 (10)	0.85695 (10)	0.0357 (3)	
C12	−0.4875 (2)	0.51625 (10)	0.80224 (11)	0.0383 (3)	
C13	−0.6624 (3)	0.47198 (12)	0.82474 (13)	0.0490 (4)	
H13	−0.7045	0.4263	0.7891	0.059*	
C14	−0.7732 (3)	0.49541 (13)	0.89940 (15)	0.0566 (5)	
H14	−0.8905	0.4657	0.9138	0.068*	
C15	−0.7125 (3)	0.56253 (13)	0.95334 (14)	0.0584 (5)	
H15	−0.7889	0.5782	1.0036	0.070*	
C16	−0.5388 (3)	0.60604 (12)	0.93243 (12)	0.0474 (4)	
H16	−0.4979	0.6511	0.9691	0.057*	
S1	0.18651 (6)	0.61737 (3)	0.51673 (2)	0.03890 (13)	
N1	0.42477 (18)	0.64443 (9)	0.53490 (9)	0.0382 (3)	
N2	0.03875 (19)	0.67010 (8)	0.76209 (9)	0.0378 (3)	
N3	−0.12812 (19)	0.61537 (8)	0.76789 (9)	0.0374 (3)	
O1	0.06395 (19)	0.68861 (10)	0.49308 (9)	0.0581 (4)	
O2	0.1846 (2)	0.54798 (10)	0.45479 (9)	0.0610 (4)	
O3	−0.3885 (2)	0.49217 (9)	0.72635 (9)	0.0557 (4)	
H3	−0.285 (4)	0.5240 (15)	0.7235 (16)	0.067*	

### Atomic displacement parameters (Å^2^)

**Table:**

	*U*^11^	*U*^22^	*U*^33^	*U*^12^	*U*^13^	*U*^23^
C1	0.0359 (8)	0.0874 (14)	0.0524 (11)	−0.0090 (9)	0.0175 (7)	−0.0246 (10)
C2	0.0275 (6)	0.0353 (7)	0.0342 (7)	−0.0008 (5)	0.0019 (5)	0.0000 (6)
C3	0.0329 (7)	0.0478 (9)	0.0448 (9)	−0.0081 (6)	0.0076 (6)	−0.0008 (7)
C4	0.0379 (8)	0.0441 (9)	0.0537 (10)	−0.0121 (7)	0.0005 (7)	0.0002 (8)
C5	0.0475 (9)	0.0395 (8)	0.0437 (9)	−0.0083 (7)	−0.0031 (7)	−0.0046 (7)
C6	0.0411 (8)	0.0380 (8)	0.0363 (8)	−0.0042 (6)	0.0048 (6)	−0.0022 (6)
C7	0.0294 (6)	0.0327 (7)	0.0335 (7)	−0.0012 (5)	0.0025 (5)	0.0028 (6)
C8	0.0323 (7)	0.0351 (7)	0.0297 (7)	−0.0033 (6)	0.0024 (5)	0.0026 (6)
C9	0.0585 (10)	0.0508 (10)	0.0323 (8)	−0.0227 (8)	0.0126 (7)	−0.0050 (7)
C10	0.0363 (7)	0.0381 (8)	0.0383 (8)	−0.0018 (6)	0.0072 (6)	0.0000 (6)
C11	0.0341 (7)	0.0371 (8)	0.0362 (7)	0.0011 (6)	0.0087 (6)	0.0045 (6)
C12	0.0389 (8)	0.0383 (8)	0.0381 (8)	0.0004 (6)	0.0059 (6)	0.0039 (6)
C13	0.0463 (9)	0.0464 (9)	0.0543 (10)	−0.0108 (8)	0.0046 (8)	0.0018 (8)
C14	0.0418 (9)	0.0618 (12)	0.0668 (12)	−0.0125 (8)	0.0164 (8)	0.0091 (10)
C15	0.0516 (10)	0.0650 (12)	0.0598 (11)	−0.0021 (9)	0.0295 (9)	0.0004 (10)
C16	0.0485 (9)	0.0491 (10)	0.0453 (9)	−0.0028 (8)	0.0171 (7)	−0.0049 (7)
S1	0.03155 (19)	0.0558 (3)	0.0296 (2)	−0.01232 (16)	0.00564 (13)	−0.00418 (16)
N1	0.0279 (6)	0.0499 (8)	0.0371 (7)	−0.0062 (5)	0.0078 (5)	−0.0085 (6)
N2	0.0333 (6)	0.0401 (7)	0.0403 (7)	−0.0054 (5)	0.0083 (5)	−0.0013 (5)
N3	0.0331 (6)	0.0396 (7)	0.0397 (7)	−0.0053 (5)	0.0084 (5)	0.0011 (5)
O1	0.0377 (6)	0.0885 (10)	0.0479 (7)	0.0080 (6)	−0.0021 (5)	0.0094 (7)
O2	0.0638 (8)	0.0775 (9)	0.0423 (7)	−0.0335 (7)	0.0167 (6)	−0.0228 (6)
O3	0.0623 (8)	0.0551 (8)	0.0505 (7)	−0.0147 (6)	0.0214 (6)	−0.0147 (6)

### Geometric parameters (Å, º)

**Table:**

C1—N1	1.4641 (19)	C9—H9B	0.9700
C1—H1A	0.9600	C10—N3	1.286 (2)
C1—H1B	0.9600	C10—C11	1.449 (2)
C1—H1C	0.9600	C10—H10	0.9300
C2—C3	1.394 (2)	C11—C16	1.399 (2)
C2—C7	1.4104 (19)	C11—C12	1.400 (2)
C2—N1	1.4203 (19)	C12—O3	1.349 (2)
C3—C4	1.376 (2)	C12—C13	1.389 (2)
C3—H3A	0.9300	C13—C14	1.372 (3)
C4—C5	1.379 (2)	C13—H13	0.9300
C4—H4	0.9300	C14—C15	1.378 (3)
C5—C6	1.377 (2)	C14—H14	0.9300
C5—H5	0.9300	C15—C16	1.371 (3)
C6—C7	1.396 (2)	C15—H15	0.9300
C6—H6	0.9300	C16—H16	0.9300
C7—C8	1.4745 (19)	S1—O2	1.4242 (13)
C8—N2	1.2870 (19)	S1—O1	1.4252 (15)
C8—C9	1.504 (2)	S1—N1	1.6358 (13)
C9—S1	1.7519 (16)	N2—N3	1.4004 (17)
C9—H9A	0.9700	O3—H3	0.85 (2)
			
N1—C1—H1A	109.5	N3—C10—C11	122.22 (15)
N1—C1—H1B	109.5	N3—C10—H10	118.9
H1A—C1—H1B	109.5	C11—C10—H10	118.9
N1—C1—H1C	109.5	C16—C11—C12	118.29 (14)
H1A—C1—H1C	109.5	C16—C11—C10	119.41 (15)
H1B—C1—H1C	109.5	C12—C11—C10	122.30 (14)
C3—C2—C7	119.28 (14)	O3—C12—C13	117.56 (16)
C3—C2—N1	118.96 (13)	O3—C12—C11	122.50 (14)
C7—C2—N1	121.73 (13)	C13—C12—C11	119.93 (15)
C4—C3—C2	120.75 (15)	C14—C13—C12	120.17 (17)
C4—C3—H3A	119.6	C14—C13—H13	119.9
C2—C3—H3A	119.6	C12—C13—H13	119.9
C3—C4—C5	120.71 (15)	C13—C14—C15	120.74 (16)
C3—C4—H4	119.6	C13—C14—H14	119.6
C5—C4—H4	119.6	C15—C14—H14	119.6
C6—C5—C4	119.02 (15)	C16—C15—C14	119.59 (17)
C6—C5—H5	120.5	C16—C15—H15	120.2
C4—C5—H5	120.5	C14—C15—H15	120.2
C5—C6—C7	122.12 (15)	C15—C16—C11	121.26 (17)
C5—C6—H6	118.9	C15—C16—H16	119.4
C7—C6—H6	118.9	C11—C16—H16	119.4
C6—C7—C2	118.11 (13)	O2—S1—O1	117.53 (9)
C6—C7—C8	119.44 (13)	O2—S1—N1	107.57 (8)
C2—C7—C8	122.44 (13)	O1—S1—N1	111.25 (8)
N2—C8—C7	118.66 (13)	O2—S1—C9	110.74 (8)
N2—C8—C9	123.59 (13)	O1—S1—C9	108.58 (9)
C7—C8—C9	117.74 (13)	N1—S1—C9	99.72 (8)
C8—C9—S1	110.29 (11)	C2—N1—C1	120.96 (13)
C8—C9—H9A	109.6	C2—N1—S1	117.24 (10)
S1—C9—H9A	109.6	C1—N1—S1	116.01 (11)
C8—C9—H9B	109.6	C8—N2—N3	114.62 (13)
S1—C9—H9B	109.6	C10—N3—N2	112.24 (13)
H9A—C9—H9B	108.1	C12—O3—H3	105.7 (16)
			
C7—C2—C3—C4	0.0 (2)	C11—C12—C13—C14	1.3 (3)
N1—C2—C3—C4	−178.31 (15)	C12—C13—C14—C15	−0.4 (3)
C2—C3—C4—C5	−0.5 (3)	C13—C14—C15—C16	−0.4 (3)
C3—C4—C5—C6	0.8 (3)	C14—C15—C16—C11	0.3 (3)
C4—C5—C6—C7	−0.6 (3)	C12—C11—C16—C15	0.6 (3)
C5—C6—C7—C2	0.1 (2)	C10—C11—C16—C15	179.69 (18)
C5—C6—C7—C8	178.70 (15)	C8—C9—S1—O2	171.30 (12)
C3—C2—C7—C6	0.2 (2)	C8—C9—S1—O1	−58.26 (15)
N1—C2—C7—C6	178.42 (14)	C8—C9—S1—N1	58.19 (14)
C3—C2—C7—C8	−178.33 (14)	C3—C2—N1—C1	−1.5 (2)
N1—C2—C7—C8	−0.1 (2)	C7—C2—N1—C1	−179.75 (16)
C6—C7—C8—N2	8.8 (2)	C3—C2—N1—S1	−153.63 (13)
C2—C7—C8—N2	−172.73 (14)	C7—C2—N1—S1	28.14 (19)
C6—C7—C8—C9	−169.98 (15)	O2—S1—N1—C2	−169.05 (12)
C2—C7—C8—C9	8.5 (2)	O1—S1—N1—C2	60.90 (13)
N2—C8—C9—S1	141.30 (14)	C9—S1—N1—C2	−53.51 (14)
C7—C8—C9—S1	−40.03 (19)	O2—S1—N1—C1	37.45 (16)
N3—C10—C11—C16	−178.83 (16)	O1—S1—N1—C1	−92.59 (15)
N3—C10—C11—C12	0.2 (2)	C9—S1—N1—C1	152.99 (15)
C16—C11—C12—O3	177.69 (16)	C7—C8—N2—N3	−178.86 (12)
C10—C11—C12—O3	−1.4 (2)	C9—C8—N2—N3	−0.2 (2)
C16—C11—C12—C13	−1.4 (2)	C11—C10—N3—N2	179.66 (14)
C10—C11—C12—C13	179.55 (15)	C8—N2—N3—C10	178.59 (14)
O3—C12—C13—C14	−177.81 (18)		

### Hydrogen-bond geometry (Å, º)

**Table:**

*D*—H···*A*	*D*—H	H···*A*	*D*···*A*	*D*—H···*A*
O3—H3···N3	0.85 (2)	1.89 (2)	2.6574 (18)	151 (2)
C1—H1*B*···O3^i^	0.96	2.59	3.534 (3)	166
C3—H3*A*···O1^ii^	0.93	2.60	3.396 (2)	144
C9—H9*A*···O2^i^	0.97	2.44	3.138 (2)	129
C16—H16···O1^iii^	0.93	2.60	3.440 (2)	151

Symmetry codes: (i) −*x*, −*y*+1, −*z*+1; (ii) *x*+1, *y*, *z*; (iii) *x*−1/2, −*y*+3/2, *z*+1/2.
